# Developmental changes in upper limb muscle synergies during throwing: A comparison between preschoolers and schoolers

**DOI:** 10.1016/j.isci.2025.113497

**Published:** 2025-09-03

**Authors:** Hiroki Saito, Ayane Kusafuka, Taishi Okegawa, Saki Takao, Naotsugu Kaneko, Hikaru Yokoyama, Ken Takiyama, Kenji Takaki, Kimitaka Nakazawa

**Affiliations:** 1 Department of Physical Therapy, Tokyo University of Technology, Tokyo, Japan; 2 Center for Human Movement, Tokyo University of Technology, Tokyo, Japan; 3 Graduate School of Arts and Sciences, Department of Life Sciences, The University of Tokyo, Tokyo, Japan; 4 Department of Intermedia Art and Science, Faculty of Science and Engineering, Waseda University, Tokyo, Japan; 5 Institute of Engineering, Tokyo University of Agriculture and Technology, Tokyo, Japan

**Keywords:** health sciences, biological sciences

## Abstract

Throwing is a fundamental motor behavior that undergoes marked refinement during childhood, yet the neuromuscular mechanisms underlying this process remain unclear. We compared upper limb muscle synergies in preschool-aged (3–5 years; *n* = 13) and school-aged (6–9 years; *n* = 8) children during overarm throwing. Participants threw balls toward a fixed target while electromyographic activity from 16 upper limb muscles and ball kinematics were recorded. Muscle synergies, extracted using non-negative matrix factorization, were analyzed for structure and sparseness. School-aged children exhibited higher ball velocity, improved accuracy, and greater synergy sparseness, reflecting more selective muscle recruitment. Cluster analysis identified eight synergy clusters in schoolers versus six in preschoolers, with several preschooler synergies fractionated into functionally specialized components in schoolers. These refinements—across the upper limb—likely enhance energy transfer efficiency and release control. The findings highlight fractionation-driven specialization as a key developmental adaptation that supports improved throwing performance.

## Highlights

• School-aged children throw a ball with greater velocity and accuracy than preschoolers
• Synergy sparseness increases with age, indicating more selective muscle recruitment
• Preschooler synergies fractionate into specialized patterns in school-aged children
• Refinements across the upper limb enhance energy transfer and release control

## Graphical abstract

**Figure:**
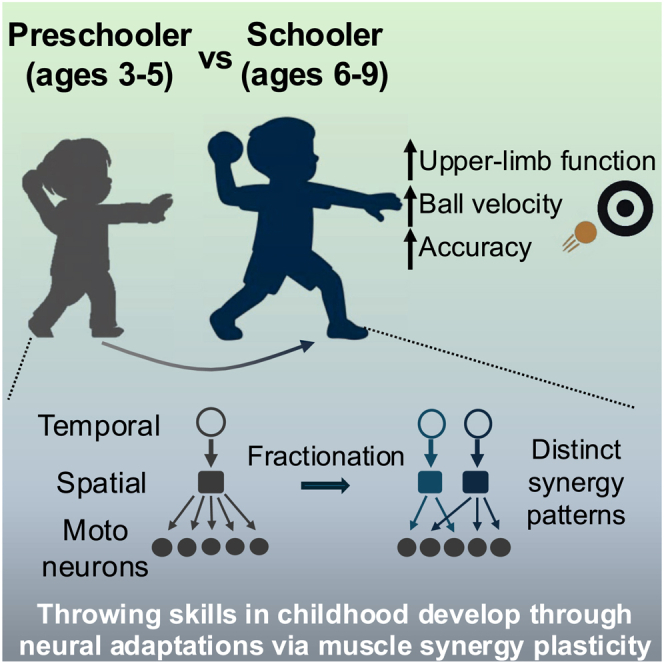

## Introduction

Throwing ability is deeply rooted in early human history, where hominins likely relied heavily on throwing projectiles to secure food and defend their resources.^1^ Over time, as tools such as the bow and arrow, nets, and firearms developed, the reliance on throwing for survival diminished.^2^ In modern society, the mechanics of throwing—central to hunting and defense for early humans—continued to be utilized, especially in sports such as baseball, cricket, and tennis.^2^ The persistence of the human ability and inclination to throw potentially accounts for its classification as an innate motor behavior that emerges early in childhood and progressively improves throughout development, even without specific training.^3^^,^^4^

The acquisition of precise throwing performance, which involves the coordinated and sequenced activation of many muscles, depends on movement patterns shaped by various factors during development.^3^ In this context, the central nervous system (CNS) plays a critical role by continually adjusting motor commands to accommodate neural constraints imposed by changes in the neuro-musculoskeletal system, such as muscle strength, muscle stiffness, and gains in neuromotor reflexes,^5^ as well as the complexity of the task, which requires precise timing, force generation, and proper sequencing of muscle activation.^6^ Therefore, the CNS must flexibly regulate the neuronal networks responsible for generating motor output to adapt to the ever-changing neuro-musculoskeletal system and the demands of a task throughout development. It has been suggested that these neuronal networks rely on a modular architecture, known as motor primitives or motor modules, which facilitates the coordination and execution of diverse motor behaviors involving thousands of motor units distributed throughout the limbs.^7^ Recent studies have indicated that some motor modules have structures that are either inborn^8^ or determined very early in life,^9^ many of which remain relatively invariant over most of the lifespan.^8^^,^^9^^,^^10^ However, it was also suggested that motor modules are newly generated or modified to meet the specific mechanical demands for the new and complex skills.^11^^,^^12^^,^^13^^,^^14^

To explore the plasticity of the neural constraints on throwing movements due to development, we focused on the developmental phase in which throwing, as a fundamental movement, is expected to improve significantly. Particularly, at a younger age such as preschoolers (3–6 years), children show a low probability of demonstrating a mature pattern of throwing, while there is rapid improvement up to middle school ages (9–10 years).^3^ Regarding the representation of motor modules, there is substantial evidence in human and animal models suggesting that a motor module can be represented as a muscle synergy in which each muscle co-activates as a single unit to circumvent the computational complexities in executing movements.^15^^,^^16^ Applying the non-negative matrix factorization (NMF) algorithm to electromyographic (EMG) data, which records activities of the muscles resulting from motoneuronal activations, identifies muscle synergies.^7^^,^^16^ Importantly, during development, synergies are shared, fractioned, and merged to adapt to various biomechanical demands and facilitate effective movement execution.^11^^,^^17^ This reorganization is driven by the plasticity of neural networks, particularly within subcortical circuits,^11^^,^^18^ and is hypothesized to result in more coordinated and effective throwing patterns.

The current study aims to examine how the NMF-derived muscle synergies during throwing differ between preschoolers and schoolers to understand the changes in neural constraints imposed by motor development in the upper limbs. We hypothesize that muscle synergies during throwing are retained and modified to reflect the differences in the competence of throwing performances.

## Results

**Figure 1:**
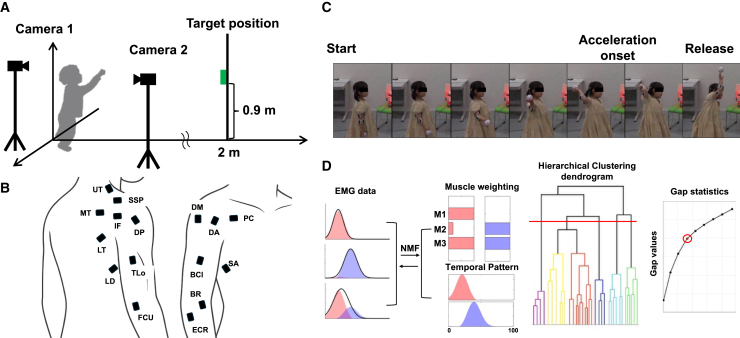
A schematic of experimental design (A) Experimental setup used in this study. Body and ball movements were recorded using two high-speed cameras placed on the participant’s back and side. The target position (0.2 m wide and long) was aligned 2 m from the throwing start position. The center of the target was located 0.9 m above the ground. (B) EMG electrode placement. The following abbreviations refer to individual muscles: IF, infraspinatus; SSP, supraspinatus; LT, lower trapezius; MT, middle trapezius; UT, upper trapezius; DA, anterior deltoid; DM, middle deltoid; DP, posterior deltoid; SA, serratus anterior; PC, pectoralis major; BCl, long head of the biceps brachii; TLo, long head of the triceps brachii; BR, brachioradialis; FCU, flexor carpi ulnaris; ECR, extensor carpi radialis longus; and LD, latissimus dorsi. (C) Sequential images of the throwing motion were captured using a high-speed camera. Three key time points—initiation, acceleration start, and release—were identified. The initiation time was determined by visual inspection as the moment when the thrower’s arm began to move upward in preparation for the cocking phase. The acceleration start time was defined as the point at which the ball’s anterior-posterior velocity reached 50% of its release velocity. The release time was defined as the moment the ball left the thrower’s hand. (D) Muscle synergy extraction using non-negative matrix factorization (NMF). Pre-processed EMG data were decomposed into time-invariant weighting components and time-varying temporal pattern components using NMF. The number of synergies was determined for each participant based on the variance accounted for (VAF), with a threshold of VAF >0.9. Determination of representative muscle synergies using hierarchical clustering and the gap statistic. Muscle weighting components extracted from individual EMG data were grouped using hierarchical clustering (Ward’s method, Euclidean distance). The optimal number of clusters was selected based on the gap statistic, which compares the compactness of actual clustering against that of reference datasets. The red dashed line on the dendrogram indicates the cut-off corresponding to the optimal number of clusters, as determined by the first local maximum of the gap statistic (highlighted with a red circle).

To clarify developmental differences in upper limb muscle synergies during throwing, preschoolers (ages 3–5) and school-aged children (ages 6–9) threw a ball toward a fixed target placed 2 m away during a standardized throwing task, and EMG activity was recorded from 16 upper limb muscles (Figures 1A and 1B). Ball velocity and accuracy were quantified based on three-dimensional trajectories reconstructed from high-speed video using DeepLabCut, a deep learning-based markerless tracking method.^19^ We first compared demographic characteristics, functional skills, and throwing performance between groups.

As shown in Table 1, Schoolers showed significantly higher scores on Pediatric Evaluation of Disability Inventory, the functional skills subscale, Part 1 (PEDI-FSS-SC1)^20^ compared to preschoolers (median [minimum–maximum]: 71 [68–72] vs. 66 [49–72], *p* = 0.004, effect size = 1.26). Additionally, schoolers exhibited superior throwing performance, characterized by higher ball velocity (6.14 [5.56–7.86] m/s vs. 4.72 [3.28–7.78] m/s, *p* = 0.022, effect size = 1.10), and greater accuracy, reflected in smaller vertical (0.04 [0.002–0.12] m vs. 0.23 [0.007–1.04] m, *p* = 0.010, effect size = 1.07) and absolute distance errors (0.18 [0.12–0.52] m vs. 0.43 [0.15–1.10] m, *p* = 0.006, effect size = 1.22). However, horizontal accuracy did not significantly differ between groups (*p* = 0.261). Representative three-dimensional ball trajectories indicated notably larger vertical and lateral movements among schoolers (Figure S1). Lastly, timing of throwing phases did not significantly differ between groups, with similar durations from movement initiation to acceleration onset (*p* = 0.962) and from acceleration onset to ball release (*p* = 0.163).

**Table 1:** Characteristics of preschoolers and schoolers

	Preschoolers (*n* = 13)	Schoolers (*n* = 8)	*p* values	Effect size
Ages (years)	4.6 (3.6–5.5)	7.2 (6.0–9.1)	*p* < 0.001	3.29
Height (cm)	1.04 (0.90–1.11)	1.17 (1.12–1.40)	*p* < 0.001	2.22
Weight (kg)	17.0 (13.4–22.2)	21.6 (18.0–38)	*p* < 0.001	1.59
Gender (male/female)	5/8	4/4	–	–
PEDI-FSS-SC1	66 (49–72)	71 (68–72)	*p* = 0.004	1.26
Ball-velocity (m/s)	4.72 (3.28–7.78)	6.14 (5.56–7.86)	*p* = 0.022	1.10
Ball-accuracy (horizontal) (m)	0.11 (0.008–0.251)	0.07 (0.007–0.22)	*p* = 0.261	–
Ball-accuracy (vertical) (m)	0.23 (0.007–1.04)	0.04 (0.002–0.12)	*p* = 0.010	1.07
Ball-accuracy (absolute) (m)	0.43 (0.15–1.10)	0.18 (0.12–0.52)	*p* = 0.006	1.22
Duration from start of movement to acceleration onset (s)	1.02 (0.92–1.38)	1.17 (0.84–1.39)	*p* = 0.962	–
Duration from acceleration onset to release (s)	0.06 (0.02–0.09)	0.07 (0.06–0.10)	*p* = 0.163	–

### Representative muscle synergies in preschoolers and schoolers

**Figure 2:**
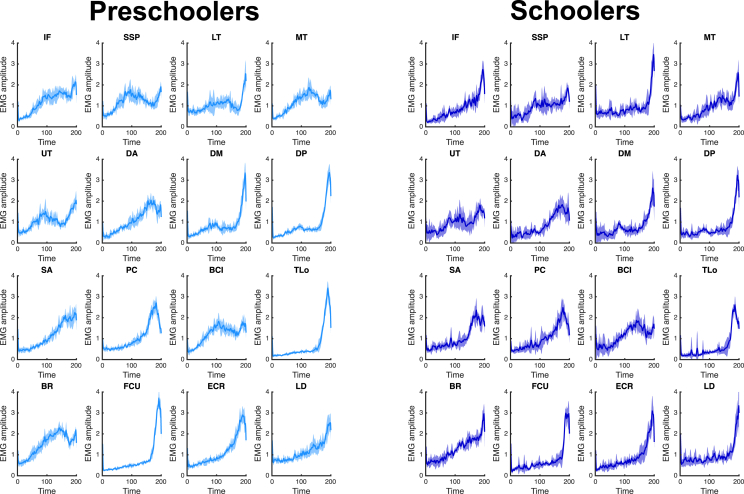
Electromyographic signals in preschoolers and schoolers during throwing Electromyographic (EMG) signals were recorded from 16 upper limb muscles during five successful repetitions. The mean EMG envelope across all participants is shown as a line, with the standard deviation represented as a shaded area around it. Signal amplitudes were normalized to the maximum value of each muscle during the task and standardized to unit variance. IF, infraspinatus; SSP, supraspinatus; LT, lower trapezius; MT, middle trapezius; UT, upper trapezius; DA, anterior deltoid; DM, middle deltoid; DP, posterior deltoid; SA, serratus anterior; PC, pectoralis; BCl, long head of the biceps; TLo, long head of the triceps; BR, brachioradialis; FCU, flexor carpi ulnaris, ECR, extensor carpi radialis longus; LD, latissimus dorsi.

**Figure 3:**
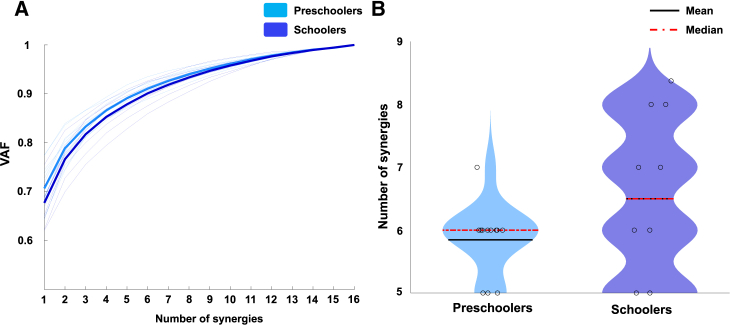
Variance accounted for (VAF) and the number of synergies n preschoolers and schoolers (A) VAF by each muscle synergy in preschoolers and schoolers. Individual (thin lines) and overall group (thick lines) means of VAF are presented for each group. (B) The number of muscle synergies in preschoolers and schoolers. The black line represents the means, and median values are indicated by red dotted lines. No significant differences were observed in the number of synergies between the two groups (median [range]: 6.0 [5–7] vs. 6.5 [5–8], *p* = 0.075).

The acquired EMG data were rectified and then filtered with a low-pass filter to smooth the signal (Figure 2). Next, muscle synergies were extracted using non-negative matrix factorization (NMF) for each participant.^21^^,^^22^ Figure 3A shows the variance accounted for (VAF), and Figure 3B shows the number of muscle synergies for preschoolers and schoolers. No significant differences were observed in the number of synergies between the two groups (6.0 [5–8] vs. 6.5 [5–8], *p* = 0.574).

**Figure 4:**
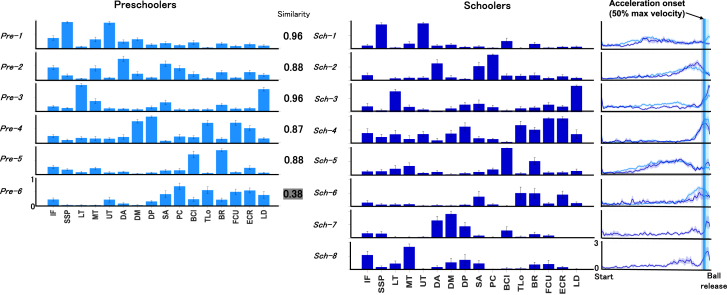
Representative shared and specific muscle synergies in preschoolers and schoolers Representative muscle synergies were identified using hierarchical cluster analysis (Ward’s method, Euclidean distance). The optimal number of clusters was determined by the gap statistic. Representative muscle synergies were identified using hierarchical cluster analysis (Ward’s method, Euclidean distance). The optimal number of clusters was determined by the gap statistic. The bars represent the mean of weighting components in each cluster, and the error bars indicate the standard error (SE). The corresponding temporal patterns are also shown, where the mean is plotted as a line and the SE is shaded around the line. IF, infraspinatus; SSP, supraspinatus; LT, lower trapezius; MT, middle trapezius; UT, upper trapezius; DA, anterior deltoid; DM, middle deltoid; DP, posterior deltoid; SA, serratus anterior; PC, pectoralis; BCl, long head of the biceps; TLo, long head of the triceps; BR, brachioradialis; FCU, flexor carpi ulnaris; ECR, extensor carpi radialis longus; LD, latissimus dorsi.

Representative muscle synergies identified via hierarchical cluster analysis are illustrated in Figure 4, encompassing both muscle weighting and temporal pattern components. A total of six synergy clusters were extracted for preschoolers and eight for schoolers. Among these, five clusters were shared between groups, showing high similarity in muscle weighting components (scalar product [SP] > 0.75), indicating common neuromuscular coordination strategies. Conversely, one synergy was unique to schoolers, and three were specific to preschoolers (SP < 0.75). Temporal patterns of these synergies were compared using statistical parametric mapping (SPM; spm1d v0.4.7 for MATLAB), and no significant differences were found between the groups.

### Fractionation of muscle synergies associated with development

**Figure 5:**
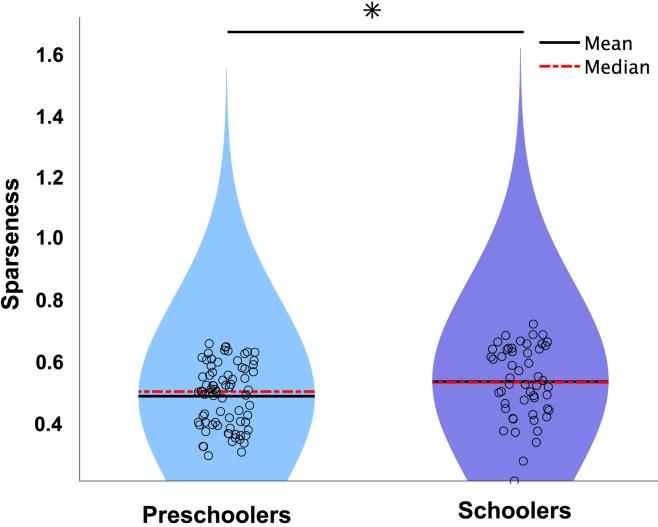
Muscle synergy sparseness in preschoolers and schoolers Synergy sparseness, calculated from each synergy vector as an index of how selectively muscle activity is distributed across muscles (higher values indicate activation of fewer, more specific muscles; lower values indicate broader co-activation), was compared between groups. The black line represents the means, and median values are indicated by red dotted lines. School-aged children exhibited significantly greater sparseness than preschoolers, reflecting more functionally specialized muscle recruitment (median [range]: 0.53 [0.20–0.72] vs. 0.50 [0.29–0.65], *p* = 0.013, d = 0.43). Asterisks indicate statistical significance (*p* < 0.05; Mann-Whitney U test).

To investigate developmental changes in the structure of muscle synergies, we assessed the sparseness of synergy vectors, which reflects the selectivity of muscle recruitment within each synergy.^11^ As shown in Figure 5, synergy sparseness was significantly higher in schoolers than in preschoolers (0.53 [0.20–0.72] vs. 0.50 [0.29–0.65], *p* = 0.013, d = 0.43), indicating that school-aged children tend to activate more functionally specific muscle groups, whereas younger children exhibit broader co-contractions.

**Figure 6:**
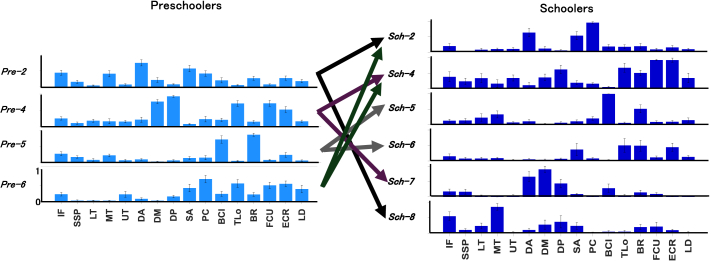
Representative muscle synergies in schoolers explained by the fractionation of preschooler synergies Each preschooler synergy fractionates into two distinct synergies observed in schoolers: Pre-2 into Sch-2 and Sch-8 (fractionation coefficients: 0.70 and 0.40), Pre-4 into Sch-4 and Sch-7 (coefficients: 0.72 and 0.35), Pre-5 into Sch-5 and Sch-6 (coefficients: 0.78 and 0.27), and Pre-6 into Sch-2 and Sch-4 (coefficients: 0.49 and 0.60). The bars represent the mean of weighting components in each cluster, and the error bars indicate the standard error (SE). IF, infraspinatus; SSP, supraspinatus; LT, lower trapezius; MT, middle trapezius; UT, upper trapezius; DA, anterior deltoid; DM, middle deltoid; DP, posterior deltoid; SA, serratus anterior; PC, pectoralis; BCl, long head of the biceps; TLo, long head of the triceps; BR, brachioradialis; FCU, flexor carpi ulnaris; ECR, extensor carpi radialis longus; LD, latissimus dorsi.

To further evaluate whether this increased sparseness could be explained by synergy fractionation—a developmental process in which single synergies are divided into multiple, more specialized components—we modeled each synergy cluster centroid from preschoolers as a linear combination of two or more synergies from schoolers using non-negative least squares.^11^ A synergy was considered fractionated if the reconstruction reached SP ≥ 0.75 and the contributing schooler synergies had coefficients ≥0.2. As shown in Figure 6, representative muscle synergies in schoolers were explained by fractionation of preschooler synergies. Specifically, each preschooler synergy fractionated into two distinct synergies observed in schoolers: Pre-2 fractionated into Sch-2 and Sch-8 (fractionation coefficients: 0.70 and 0.40, respectively), Pre-4 into Sch-4 and Sch-7 (0.72 and 0.35, respectively), Pre-5 into Sch-5 and Sch-6 (0.78 and 0.27, respectively), and Pre-6 into Sch-2 and Sch-4 (0.49 and 0.60, respectively).

## Discussion

This study provides insights into the developmental plasticity of neural mechanisms underlying upper limb coordination during throwing movements from preschool (3–5 years) to school age (6–9 years). Our results indicate that improvements in throwing performance, particularly in ball velocity and accuracy (notably in the vertical direction), coincide with significant refinements in the organization of muscle synergies. Although several foundational muscle synergies (shared synergies) were consistently observed across both age groups, suggesting the early emergence and preservation of basic neural control strategies, schoolers demonstrated increased synergy sparseness and fractionation. These observed changes reflect a developmental trajectory toward more selective and functionally distinct muscle activation patterns, supporting enhanced motor performance during childhood.

### Foundational motor patterns of throwing in preschoolers and schoolers

First, we found that five synergies were similar between preschoolers and schoolers (SP > 0.8), suggesting a foundational neuromuscular control strategy for throwing, characterized by the coordinated activation of various upper limb muscles. Pre-1 and Sch-1 represent the activations of supraspinatus (SSP) and upper trapezius (UT) to abduct shoulder. Pre-2 and Sch-2, with activations of anterior deltoid (DA), serratus anterior (SA), and pectoralis (PC), is largely activated before the acceleration onset. This is followed by Pre-3 and Sch-3 with the activations of lower trapezius (LT), middle trapezius (MT), and latissimus dorsi (LD), which can act for scapular upward rotation and stability, it contributed to foundational scapulohumeral rhythm to further raise arm to prepare release.^23^ This is followed by Pre-4 and Sch-4 in long head of the triceps (TLo) and deltoid muscle (DM and DP) together with wrist muscles to keep holding a ball to for the release. In addition, Pre-5 and Sch-5 are characterized by the activation of long head of the biceps (BCl) and brachioradialis (BR), which function as elbow flexors associated with the cocking phase, helping to maintain the arm in the overhead position. Overall, these patterns are consistent with the general idea that foundational sequence patterns in overhead throwing occur proximal to distal for effectively transferring the kinetic energy to a ball.^24^ These seemingly sophisticated sequential patterns in both preschoolers and schoolers, despite the lack of prior specific throwing training, suggest that the motor patterns involved in throwing may result from early natural learning, environmental influences, or maybe primarily rooted in inborn primitives, similar to locomotor control.^8^

### Developmental changes in schooler’s synergies related to increased throwing performance

Although the total number of muscle synergies identified did not significantly differ between preschoolers and schoolers, the cluster analysis revealed greater complexity in the synergy organization among schoolers, with eight distinct synergy clusters compared to six in preschoolers, including three unique clusters specific to schoolers. Furthermore, the synergy compositions were significantly sparser in schoolers, indicating a developmental shift toward more selective and functionally specialized muscle activation patterns.

Specifically, we identified a clear fractionation of muscle synergies from preschoolers into more distinct patterns observed in schoolers (Figure 5). This fractionation likely represents a refinement in neuromuscular control, aligning with improved motor performance as reflected by increased throwing velocity and accuracy observed among schoolers.^18^ Four distinct preschooler synergy clusters (Pre-2, Pre-4, Pre-5, and Pre-6) fractionated into two discrete clusters each in the schooler group, reflecting a reduction in joint co-activation and an enhancement of specific functional roles for each joint (Figure 6). For instance, preschoolers’ synergy cluster Pre-2, characterized by co-activations of infraspinatus (IF), MT, DA, SA, and PC, fractionated into two functionally specialized synergies in schoolers: Sch-2 (dominated by DA, SA, and PC) focusing on a pushing action, and Sch-8 (IF and MT dominant) emphasizing external shoulder rotation. Along with more consolidating distinct roles of pushing the arm forwards by Sch-2, we noted IF increased shoulder external rotation, allowing the thrower to generate more rotational force and providing a longer acceleration phase, which enhances control over the ball’s release and trajectory.^25^ MT related to scapular retraction, which act for stabilizing the shoulder joint and aligning the arm optimally for throwing, reduces compensatory movements and helps ensure a more efficient path toward the target, further enhancing precision.^26^ These shoulder-focused refinements observed in schoolers may explain the larger ball trajectories, particularly upward and toward the first-base direction (Figure S1), likely due to enhanced shoulder rotation functions, which increased the effectiveness of the cocking maneuver during throwing. Interestingly, the development of rotational components in the shoulder complex, which permits greater elastic energy storage and release, is a distinct feature in humans compared to their closest extant relatives, such as chimpanzees, providing a selective advantage during hunting.^2^ These neuromuscular refinements are consistent with previous research,^27^ which demonstrated that age-related advancements in throwing performance involve a developmental shift from simple, synchronized joint coordination patterns toward patterns characterized by greater diversity and variability in timing among joints.

### Neural mechanisms underlying synergy adaptation

The neural mechanisms underpinning development-related alterations of muscle synergies remain speculative. Muscle synergies are instantiated through complex neural networks distributed across multiple levels of the CNS, including cortical motor areas, brainstem nuclei, and spinal premotor interneurons.^18^ In particular, experimental evidence has highlighted pivotal contributions from spinal interneuronal circuits, which exhibit divergent projections onto motoneuron pools, forming fundamental modules for coordinated muscle activations.^28^ The primary motor cortex recruits or modulates downstream neurons that either organize entire muscle synergies or contribute as part of the synergy-organizing networks, especially in the upper limb movements.^18^^,^^29^ In early developmental stages, due to immature sensory and/or descending modulation from cortical regions, the CNS relies predominantly on spinal and brainstem circuits that provide primitive coordination.^8^^,^^30^^,^^31^ As children grow, the gradual maturation of anthropometric and other neuro-musculoskeletal properties enhances sensory signaling. Furthermore, the corticospinal tract, responsible for primary voluntary motor commands from the brain to the spinal cord, continues to mature throughout childhood, involving processes such as myelination and synaptic refinement.^32^^,^^33^ This maturation enables distinct control of muscle subgroups within the original synergies.^11^^,^^34^

A previous study proposed that a potential mechanism underlying synergy fractionation is the recruitment of additional neural circuits by the CNS to subdivide control.^11^ Specifically, if a previously inactive or “idle” spinal circuit becomes available, descending pathways from the brain can repurpose this circuit, allocating it to control a subset of muscles previously co-activated by another synergy. With repeated activation through differentiated timing signals, these new neural connections become strengthened. Critically, this refinement process is thought to be orchestrated by supraspinal centers—including motor cortical areas—which modulate the activity and synchronization of spinal interneuronal circuits to achieve fine motor control.^11^ By the end of typical motor development, muscle synergies tend to consolidate into sparse, functionally and anatomically closed groups.^35^ These refined synergies serve as minimal functional units that can be flexibly recombined, providing a neural substrate for the rich variety of goal-directed motor behaviors seen in humans.^13^^,^^14^

### Clinical implication

In our study, school-age children achieved significantly higher ball velocities and better throwing accuracy than preschoolers, and these improvements corresponded with their more refined synergy organization. A less fractionated, co-contraction-based synergy pattern in younger children tends to limit performance: co-contraction stiffens the body and can hinder the generation of fast movements. When too many muscles are bound into a single synergy, movement becomes low-dimensional and less adaptable—an issue observed in neurological impairments such as stroke and spinal cord injuries, where merging of synergies (i.e., a reduced number of modules) is associated with poorer motor outcomes.^36^^,^^37^^,^^38^ By contrast, schoolers were able to activate a greater number of synergies with more distinct functional roles, potentially resulting in a more coordinated sequence of throwing—consistent with previous research.^27^ These results reinforce the idea that increasing the modular complexity of motor control is beneficial for performance. Having more distinct synergies provides the CNS with a richer “toolbox” of movement components that can be flexibly combined and adapted. Future research should investigate interventions that specifically promote increased modular complexity or optimal synergy fractionation.

### Limitations of the study

A primary limitation of this study is the absence of detailed arm kinematic data. The lack of reliable kinematic measurements significantly constrains our ability to precisely define several movement phases, such as the cocking phase, and thus limits the mechanical interpretation of muscle synergy activations. Consequently, interpretations presented in the discussion regarding muscle activations are speculative and should be viewed cautiously. Additionally, although we standardized the target height across age groups, there were inherent differences in participants’ heights between preschoolers and schoolers. This disparity potentially altered the required throwing patterns needed to accurately hit the target, possibly influencing differences in synergy recruitment between age groups.

## Resource availability

### Lead contact

Further information and requests for resources and reagents should be directed to and will be fulfilled by the lead contact, Kimitaka Nakazawa (nakazawa@idaten.c.u-tokyo.ac.jp), Department of Life Sciences, Graduate School of Arts and Sciences, The University of Tokyo, 3-8-1 Komaba, Meguro, Tokyo 153–8902, Japan.

### Materials availability

This study did not generate new unique reagents.

### Data and code availability

• Participant raw EMG data collected during throwing tasks are available at https://doi.org/10.17632/r55nj4jxt7.1.
• Code will be shared by the lead contact upon request.
• Any additional information required to reanalyze the data reported in this paper is available from the lead contact upon request.

## Acknowledgments

We would like to acknowledge the valuable assistance of the Doronko Welfare Foundation (社会福祉法人どろんこ会) for their cooperation in the recruitment of participants and for helping to facilitate the smooth execution of the experiments. This research is supported by the Nippon Professional Baseball Organization and Agekke Corporation.

## Author contributions

Conceptualization, H.S., A.K., T.O., and K.N.; methodology, H.S., A.K., T.O., N.K., and K.N.; software: H.S., A.K., T.O., and S.T; formal analysis, H.S., A.K., T.O., S.T., H.Y., K.Takiyama, and K.Takaki; investigation, H.S., A.K., T.O., N.K., and S.T.; data curation, H.S., A.K., T.O., S.T., and N.K.; writing – original draft, H.S. and A.K.; writing – review & editing, H.S., A.K., T.O., S.T., N.K., H.Y., K.Takiyama, K.Takaki, and K.N.; visualization, H.S. and A.K.; supervision, H.Y., K.Takiyama, and K.N.; funding acquisition, K.N.

## Declaration of interests

The authors declare no competing interests.

## STAR★Methods

### Key resources table

**Table:** 

REAGENT or RESOURCE	SOURCE	IDENTIFIER
MATLAB, 2024a	MathWorks	www.mathworks.com/
DeepLabCut	Mathis Laboratory	www.mackenziemathislab.org/deeplabcut
SPM	spm1d v0.4.7 for MATLAB	spm1d.org/

### Experimental model and subject details

#### Participants

Initially, 29 children participated in this study: 19 preschoolers (ages 3–5 years) and 10 school-aged children (ages 6–10 years). Three preschoolers (aged 5.7, 5.8, and 5.9 years) were excluded to establish a clearer developmental age gap between groups. Additionally, data from four participants (three preschoolers and one schooler) were excluded due to technical issues during data collection described below, and one schooler was excluded due to an insufficient number of valid throwing trials. Consequently, the final dataset for analysis included 21 participants: 13 preschoolers (median age: 4.6 years; range: 3.6–5.5 years) and 8 school-aged children (median age: 7.2 years; range: 6.0–9.1 years) shown in Tables 2 and 3

**Table:** Preschooler characteristics

Participant	Gender	Age (years)	Height (m)	Weight (kg)	PEDI-FSS-SC1
Pre1	M	3.7	1.03	17.0	63
Pre2	F	4.3	1.10	17.0	49
Pre3	F	5.5	1.11	17.8	65
Pre4	M	4.8	1.08	19.2	60
Pre5	F	4.0	0.96	15.2	63
Pre6	F	4.6	0.98	13.4	69
Pre7	M	4.8	1.05	16.6	71
Pre8	F	4.1	1.00	15.6	61
Pre9	F	3.6	0.90	18.8	66
Pre10	F	5.1	1.03	18.8	72
Pre11	F	4.6	1.01	17.0	66
Pre12	M	5.2	1.10	21.8	68
Pre13	M	5.2	1.10	22.2	69

Pre: preschoolers; PEDI-FSS-SC1, Pediatric Evaluation of Disability Inventory, the functional skills subscale, Part 1 (PEDI-FSS-SC1).

**Table:** Schooler characteristics

Participant	Gender	Age (years)	Height (m)	Weight (kg)	PEDI-FSS-SC1
Sch1	F	7.3	1.14	21.3	72
Sch2	F	9.1	1.37	38.0	72
Sch3	F	8.5	1.30	32.4	72
Sch4	F	7.1	1.14	20.4	68
Sch5	M	6.0	1.12	20.4	71
Sch6	M	6.1	1.15	18.0	70
Sch7	M	8.4	1.40	35.0	72
Sch8	M	6.6	1.18	22.0	69

Sch, schoolers; PEDI-FSS-SC1, Pediatric Evaluation of Disability Inventory, the functional skills subscale, Part 1 (PEDI-FSS-SC1).

.

The study was conducted in accordance with the Declaration of Helsinki. All procedures were approved by the local committee in the University of Tokyo (reference number: 764). The parents or guardians of all participating preschoolers and school-aged children provided informed consent prior to the start of the study.

### Method details

#### Experimental protocol

All experiments were conducted in an indoor laboratory. Prior to the experiment, participants performed a brief warm-up consisting of 3–5 light throws to become familiar with the task and instructions. These throws were not recorded and were solely intended to ensure task understanding and comfort. Each participant threw fastballs at a target (0.2 m × 0.2 m) marked on a board. The board was positioned 2 m from the throwing start position, with the target center placed 0.9 m above the ground. Each participant completed 10–20 throws, and they were instructed to throw as accurately as possible at the target (Figure 1A).

#### Data collection

We assess functional skills for all participants using Pediatric Evaluation of Disability Inventory (PEDI).^20^ PEDI consists of 197 functional skills and 20 composite daily activities, all categorized into three domains: self-care, mobility, and social function.^20^ Since the study focused on the function of upper limbs, we only implemented the functional skills subscale, Part 1, in the domain of self-care (PEDI-FSS-SC1), which consisted of 73 items.^20^

Kinematic data of the ball were collected using the same method as the previous study.^39^ Ball movements were recorded using high-speed cameras (DSC-RX10M4, SONY, Japan; 960 fps) positioned to capture both back and side views of the participant. The cameras were synchronized using an LED light and a commercial synchronization system (FA-WRC1M and FA-WRR1, SONY, Japan) that enabled simultaneous shutter release. As there were no markings on the ball, the ball’s center coordinates were determined from the camera images. Data collection covered approximately 1,000 ms, spanning from the initiation of the throwing motion until 10 ms after the ball’s arrival. Data were excluded if the ball movement was outside the cameras’ field of view or if the LED light flash occurred outside the data collection phase due to inter-trial movement variability. Consequently, data from four participants were excluded, leaving 25 participants for the subsequent analyses.

EMG activity was recorded from the following 16 muscles distributed across the right side of the upper limbs (Figure 1B): infraspinatus (IF), supraspinatus (SSP), lower trapezius (LT), middle trapezius (MT), upper trapezius (UT), anterior deltoid (DA), middle deltoid (DM), posterior deltoid (DP), serratus anterior (SA), pectoralis (PC), long head of the biceps (BCl), long head of the triceps (TLo), brachioradialis (BR), flexor carpi ulnaris (FCU), extensor carpi radialis longus (ECR) and latissimus dorsi (LD). EMG activity was recorded using Pico EMG sensors (Cometa Srl, Italy). The EMG signals were sampled at 2000 Hz. The data from the high-speed cameras and EMG system were synchronized using the optical LED light and its analog signal.

### Quantification and statistical analysis

#### Kinematic variable

Ball tracking in high-speed camera images was performed using DeepLabCut,^19^ a deep learning-based automatic image recognition framework. The tracking models were trained using manually labeled images from one trial per participant. For each trial, twenty frames were automatically selected by DeepLabCut’s built-in frame extraction algorithm, which identifies optimal frames for manual labeling. This resulted in a training dataset of 340 labeled images. Separate models were developed for each camera view and trained for 35,000 to 55,000 iterations. These trained models were then used to automatically track and digitize the ball’s position in all remaining trials.

A calibration frame with 27 points was used for coordinate transformation. The points were arranged in a 3 × 3×3 grid with intervals of 0.1 m horizontally (x axis), 0.2 m vertically (z axis), and 0.1 m in the home plate-pitcher’s plate direction (y axis). The calibration points were digitized using numerical analysis software (MATLAB, Mathworks, Japan). Three-dimensional ball coordinates were reconstructed using Three-dimensional Direct Linear Transformation (3D DLT) in MATLAB. The position data were filtered using a fourth-order Butterworth low-pass digital Butterworth filter with a 60 Hz cut-off frequency. This frequency was determined through residual analysis and qualitative assessment of the velocity curves. The global coordinate system was defined with its origin at the center of the pitcher’s plate: the x axis pointed toward the third base, the y axis toward home plate, and the z axis vertically upward. Ball speed was calculated as the magnitude of the velocity vector at the moment of ball release.

The ball’s arrival position was determined by manually digitizing the ball’s center point in the back-view camera images at the moment of arrival, whether the ball hit the target board or the floor. For coordinate transformation, a calibration grid of 16 points was used, arranged in a 4 × 4 pattern. The horizontal intervals between points were 2.5 m, 2.44 m, and 2.5 m, while the vertical intervals were 0.62 m, 0.81 m, and 0.77 m. The calibration was performed using measured reference marks on the board. Two-dimensional Direct Linear Transformation (2D DLT) was then applied to calculate the ball’s arrival position coordinates. Ball accuracy was calculated as the horizontal distance, vertical distance, and mean absolute distance of the ball arrival position relative to the center of the target on the x–z plane. The tracking procedure used in this study was the same as used in previous studies using throwing tasks.^39^^,^^40^ Its accuracy has already been verified compared to conventional methods,^39^ and the absolute distance between the 3D coordinates obtained from this method and conventional method was an average of 15.5–29.4 mm, and the correlation coefficients between them ranged from 0.932 to 0.999.

Four key time points during the throwing motion were identified Figure 1C): Start time, Acceleration onset, Release, and End time. Start time was defined by visually identifying the onset of arm movement. Acceleration onset was defined as the time point at which the forward velocity of the ball reached 50% of its release velocity in each trial, calculated using the position coordinates of the ball. Ball release was determined by qualitative observation of the frame in which the ball was no longer in contact with the hand, based on footage from a camera positioned to the side of the thrower. End time was defined as the frame in which the ball made contact with either the target board or the floor.

#### EMG processing

For this study, we analyzed the EMG data from the start of the throwing motion to the moment of ball release, focusing on this specific phase. Available trial data from each participant were processed, with a median of 10 trials [5–19]. However, to mitigate potential bias due to unequal trial counts, we selected the minimum number of valid trials available across all participants—five trials per subject—for subsequent analyses. Raw analog EMG signals were high-pass filtered at 40 Hz to remove motion artifacts and demeaned.^41^ The signals were full-wave-rectified and low-pass filtered at 15 Hz, using a fourth-order Butterworth filter.^41^ The smoothed EMG envelopes were time-interpolated to generate 200 timepoints for each trial. To accurately extract muscle synergy, it is recommended to obtain the data variability in terms of muscle activations.^42^^,^^43^ Thus, we created concatenated EMG matrices from all trials available for each participant. Each EMG from each muscle was normalized to the maximum amplitude across the tasks. Then, each muscle vector in the data matrix was standardized to have unit variance so that the activity in each muscle was equally weighted.

#### Muscle synergy extraction

To explore muscle synergies, we applied non-negative matrix factorization (NMF) to the EMG matrices for each participant (Figure 1D). NMF has previously been described as a linear decomposition technique^21^^,^^22^ according to Equation 1:

(Equation 1) M=W·C+e

where *M* (*m* × *t* matrix, where *m* is the number of muscles, *t* is the number of samples (i.e., the spatiotemporal profiles of muscle activity) is a linear combination of muscle weighting components: *W* (*m* ×*n* matrix, where n is the number of muscle synergies), *C* (*n* × *t* matrix, representing temporal pattern components), and *e* is the residual error matrix. To determine the number of muscle synergies, NMF was applied to extract each possible *n* from 1 to 16 from each dataset. For temporal pattern components, since each EMG matrix contained multiple trials, the extracted temporal pattern components of were converted into an averaged trial for each participant for further analysis. The variance accounted for (VAF) by the reconstructed EMG (*M*) was calculated at each iteration to extract the optimal number of muscle synergies.^44^ VAF was defined as a 100 × square of the uncentered Pearson’s correlation coefficient.^44^^,^^45^ This subject-specific approach enables the preservation of individual differences in motor control complexity, which could otherwise be obscured by imposing a fixed number of synergies across participants.^37^ Because NMF utilizes the random initialization of W and H, the algorithm can be stuck in a suboptimal local minimum. As such, each synergy extraction was repeated 50 times, and the iteration with the highest VAF was selected.^13^^,^^14^^,^^46^^,^^47^ We utilized the criterion VAF >0.9 as a method to determine the optimal number of synergies commonly used in previous studies.^48^^,^^49^^,^^50^^,^^51^ The literature suggests that this ensures a sufficient representation of the data,^52^ although this is still debated.^53^^,^^54^

#### Muscle synergy representation

We identified a representative set of muscle synergies across preschoolers and schoolers, respectively using hierarchical clustering analysis (Ward’s method, Euclidian distance; Figure 1D).^12^ The optimal number of clusters was determined using the gap statistic,^55^ which measures the compactness of the clustering achieved against those in reference datasets without any obvious clustering similar to a previous study (Figure 1D).^11^ Reference datasets (*N* = 500) were initially generated by uniform sampling from within the bounds of the original muscle-synergy set; each of them was then clustered by the hierarchical cluster, at 2–20 clusters. The optimal number of clusters was then the smallest number, h, such that

Gap(h)≥Gap(h+1)–sd(h+1)

where Gap(k) represents the gap statistic at h clusters, and sd(h) signifies the standard deviation of the clustering compactness within the reference datasets.^55^

The muscle weighting components and their corresponding temporal pattern components within the cluster were then averaged (synergy cluster centroids). Next, we matched the relative subject-invariant cluster centroids in muscle weighting components (defined as having synergies from ≥1/3 of the participants) for between groups.^56^ Thus, we analyzed the similarity between synergy cluster centroids between groups by the scalar product between the centroids of the synergy clusters (normalized to unit vectors). For each comparison, subject-invariant cluster centroids in muscle weighting components in a group were paired with clusters in another group by maximizing the total scalar product values. Clusters that could not achieve a scalar product (SP) of 0.75 or higher were classified as unmatched.^37^^,^^57^

##### Identification of Development-Related Fractionated and Merged Synergies Using Muscle Synergy Sparseness

We explored developmental changes in muscle synergy patterns that can explain the difference in throwing performance. Previous studies have shown that synergies can be merged or fractioned to meet mechanical demands of a task through development and training specification.^11^ Thus, we explored the existence of fractionated and merged muscle synergies in schoolers that can be explained by the muscle synergies of preschoolers, associated with the development of throwing motor patterns. First, we calculated muscle synergy sparseness, which indicates the number of active muscle components within each synergy vector.^11^ Quantified the sparseness (*φ*) of each muscle synergy are the following definition:

$$\varphi =\frac{\sqrt{n}=\frac{\sum_{i=1}^{n}\left|W_{i}\right|}{\sqrt{\sum_{i=1}^{n}W_{i}^{2}}}}{\sqrt{n}-1}$$

where $W_{i}$ is the *i*th muscle component of the **W** synergy vector, and *n* = 16 is the number of muscles in the vector. According to this definition, a vector that is highly sparse with only one non-zero element has a φ value of 1, while a non-sparse vector with equal values for all muscle components has a φ value of 0. For each participant, the mean φ was determined by averaging the φ values across all of their muscle synergies. These average φ values were then compared between groups by calculating the group mean of each participant’s φ.

If the overall sparseness across all synergies is significantly greater in schoolers than in preschoolers, it suggests that the synergy in schoolers could be explained by the splitting of synergies present in preschoolers (fractionated synergies).^11^ Thus, we modeled each preschooler synergy as a linear combination of multiple fractionated synergies from schoolers^11^^,^^37^:

$$W_{i}^{b}\approx \sum_{k=1}^{N^{f}}m_{k}^{i}W_{k}^{f},m_{k}^{i}\geq 0,i=1,\ldots ,N^{f}$$

where $W_{i}^{b}$ is the *i*th centroids of the synergy cluster vectors in preschoolers to be fractioned, $W_{k}^{f}$ is the *k*th the centroids of the synergy cluster vector in schoolers fraction resulting from split, $N^{f}$ is the number of synergies that contribute to be fractioned, and *m*_*i*_ is a non-negative coefficient (fractioned coefficient) that scales the *k*th synergy in the fraction. $m_{k}^{i}$ was calculated from a non-negative least-squares fit using the lsqnonneg option in MATLAB. $W_{i}^{b}$ and $W_{k}^{f}$ were normalized as unit vectors. Synergy fractioned was identified when $N_{f}$  ≥ 2, $m_{k}^{i}$ ≥ 0.2 for all *k*, and the SP between $\sum_{k=1}^{N_{f}}m_{k}^{i}W_{k}^{f}$ and $W_{i}^{b}$ was ≥0.75.^37^^,^^57^ To investigate whether one synergy in schoolers can be explained as the fractioned synergy in preschoolers, we compared each reconstructed centroids of the synergy cluster in schoolers by every possible combination of the centroids of the synergy cluster in preschoolers.

Conversely, if the overall sparseness across all synergies is significantly lower in schoolers than in preschoolers, it suggests that the synergies in schoolers involve more co-contraction and could be explained by a merging of multiple preschooler synergies (merging synergies).^11^ Thus, we modeled the merged as a linear combination of the contributing synergies^11^^,^^37^:

$$W_{i}\approx \sum_{k=1}^{N_{b}}m_{k}^{i}W_{k}^{b},m_{k}^{i}\geq 0,i=1,\ldots ,N^{b}$$

where $W_{i}$ is the *i*th centroids of the merged synergy cluster vectors in schoolers, $W_{k}^{b}$ is the *k*th the centroids of the synergy cluster vector in schoolers to be merged, $N^{b}$ is the number of synergies that contribute to the merging, and *m*_*i*_ is a non-negative coefficient (merging coefficient) that scales the *k*th synergy in the merging. $m_{k}^{i}$ was calculated from a non-negative least-squares fit using the lsqnonneg option in MATLAB. $W_{i}$ and $W_{k}^{b}$ were normalized as unit vectors. The process of detecting instances of merging, determining the centroids of the merged synergy clusters in preschoolers, and identifying the centroid clusters representing the outcomes of these mergers was carried out using methods similar to those employed for fractionation.

#### Statistical analyses

We compared the ages, height, weight, PEDI-FSS-SC1, ball velocity, ball accuracy, the durations from start time to acceleration onset and from acceleration onset to release time, the number of synergies, and the sparseness of synergies between groups using the Mann–Whitney U test. Effect size (ES) was calculated using Cohen’s d.^58^ When temporal pattern components were matched between group, we performed statistical parametric mapping (SPM) (spm1d v0.4.7 for MATLAB) to compared the differences in the temporal patterns between groups.^59^

The significance level for all tests was set at *p* = 0.05 for all tests. We recruited 16 participants in preschoolers and 9 in schoolers without an *a priori* power analysis; we instead conducted a sensitivity analysis in G∗Power, which indicated that an effect size of 1.12 would be necessary to obtain a power of 80%.
