# Supplementary material for: Developmental changes in upper limb muscle synergies during throwing: A comparison between preschoolers and schoolers
Source: iScience. 2025 Sep 3;28(10):113497. doi: 10.1016/j.isci.2025.113497 (PMC12481115; doi:10.1016/j.isci.2025.113497)
Supplement: Document S1. Figure S1 [file mmc1.pdf]

iScience, Volume *28*

Supplemental information

Developmental changes in upper limb muscle synergies during throwing: A comparison between preschoolers and schoolers

Hiroki Saito, Ayane Kusafuka, Taishi Okegawa, Saki Takao, Naotsugu Kaneko, Hikaru Yokoyama, Ken Takiyama, Kenji Takaki, and Kimitaka Nakazawa

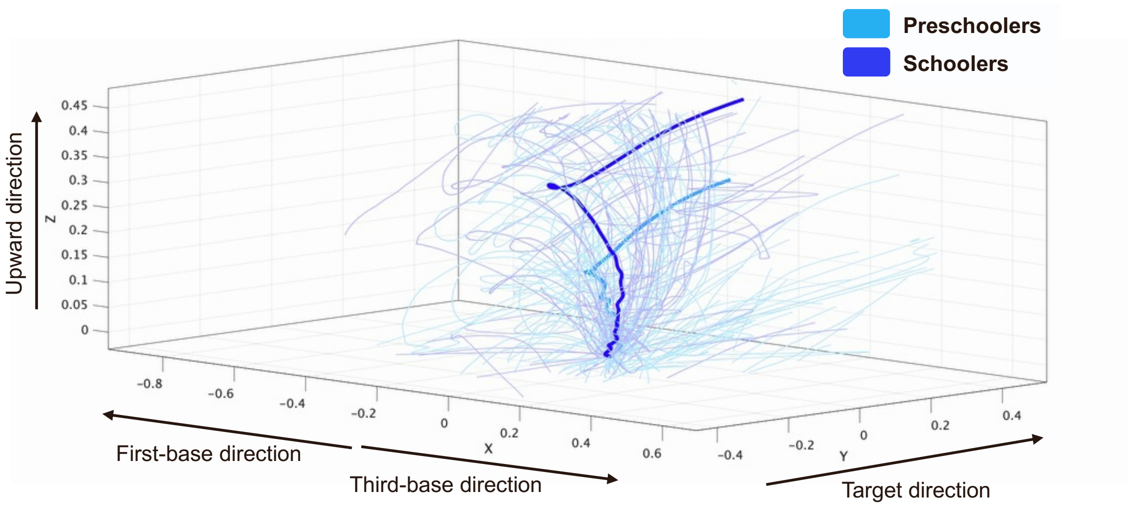

Figure S1. Representative three-dimensional trajectories of thrown balls in preschoolers and schoolers. Trajectories were obtained using synchronized high-speed cameras and reconstructed using DeepLabCut and direct linear transformation methods. The origin represents the pitching position, with axes indicating the target direction (y-axis), upward direction (z-axis), third-base direction (positive x-axis), and first-base direction (negative x-axis). The trajectories of the schoolers' throws were notably larger, particularly upward and toward the first-base direction, compared to those of preschoolers. These differences likely reflect shoulder-related developmental improvements in the cocking maneuver during throwing.
